# Spatially programmed Fe and Pt electrode-driven electrochemistry reconditions the tumor microenvironment and sensitizes triple-negative breast cancer to PD-1 Blockade

**DOI:** 10.3389/fonc.2026.1819923

**Published:** 2026-04-29

**Authors:** Jia Qin, Yi He, Yueyao Yang, Yang Jiao, Zhipeng Liu, Yijie Xie, Wanling Lu, Ming Liu, Zhengyu Zhao, Dingjun Cai, Gang Wang

**Affiliations:** 1 National Engineering Research Center for Biomaterials, College of Biomedical Engineering, Sichuan University, Chengdu, Sichuan, China; 2 Acupuncture and Tuina School, Chengdu University of Traditional Chinese Medicine, Chengdu, Sichuan, China; 3 Department of Medical Oncology/Gastric Cancer Center, West China Hospital, Sichuan University, Chengdu, Sichuan, China

**Keywords:** electrochemical therapy, ferroptosis, PD-1 blockade, pH modulation, triple-negative breast cancer, tumor microenvironment

## Abstract

Excess extracellular acidity is a defining barrier to effective antitumor immunity in solid tumors. Systemic buffers only partially correct tumor acidity and seldom rescue suppressed effector immunity. Here, we developed a spatially programmed electrochemical therapy (ECT_Fe_) integrating a central sacrificial iron anode with peripheral platinum cathodes to engineer the tumor microenvironment *in situ*. This configuration enables concurrent acidotoxic and ferroptosis-associated tumor debulking in the core, while controlled peritumoral pH relief restores immune cell function. We combined ECT_Fe_ with intramuscular delivery of a plasmid encoding anti-PD-1 single-chain antibody (pICI) for sustained checkpoint blockade. In aggressive 4T1-luc triple-negative breast cancer (TNBC), ECT_Fe_ markedly enhanced PD-1 blockade efficacy, inducing tumor regression and prolonged survival versus monotherapies. In B16 melanoma, ECT_Fe_ alone achieved robust tumor control with limited additional benefit from PD-1 blockade, revealing tumor-context-dependent interaction. Mechanistically, Fe-anode reactions generated an acidic, Fe^2+^-rich core with elevated oxidative stress and GPX4 suppression—consistent with ferroptosis—while Pt-cathode-mediated alkalinization (pH 6.6–7.0) preserved CD8^+^ T-cell activity, promoted M1-like macrophage polarization, and reduced regulatory T cells. Collectively, spatially programmed electrochemistry reconditions the tumor microenvironment to sensitize immunologically “cold” TNBC to PD-1 blockade, offering a controllable and translatable strategy to improve checkpoint immunotherapy.

## 1 Introduction

Immune checkpoint blockade (ICB) has transformed cancer therapy (1), yet a large fraction of solid tumors remains poorly responsive (2, 3). One of the major determinants of this resistance is the profoundly acidic and metabolically constrained tumor microenvironment (TME) (4). Extracellular pH levels in many tumors fall near ~6.5 (5, 6), a range that compromises cytotoxic T cell and NK cell effector functions (7–9), stabilizes regulatory T cells (Tregs) (10), and promotes the polarization of tumor-associated macrophages toward an M2-like, immunosuppressive phenotype (11, 12). These acidity-driven changes collectively blunt antitumor immunity and limit the therapeutic reach of PD-1/PD-L1 and CTLA-4 inhibitors.

Clinically, triple-negative breast cancer (TNBC) remains a challenging and often immunologically “cold” setting for immune checkpoint blockade: PD-1/PD-L1 inhibitors have shown meaningful benefit mainly in specific contexts—such as neoadjuvant chemoimmunotherapy in early-stage disease and PD-L1-positive metastatic TNBC—yet a substantial fraction of patients still does not achieve durable responses (13–15). Notably, PD-1 blockade as monotherapy yields only modest response rates in previously treated or biomarker-unselected metastatic TNBC, underscoring the importance of sensitization strategies (16). This clinical backdrop highlights the need for microenvironmental engineering strategies that can broaden and deepen ICB benefit in TNBC.

Tumor acidosis originates largely from aerobic glycolysis (the Warburg effect) (17), which generates lactate that is exported together with protons by transporters such as MCT4 and NHE1. Hypoxia-induced HIF-1α signaling further reinforces this phenotype by upregulating CAIX, NHE1, and MCT4 (18–21). High lactate not only suppresses CTL and NK cell function (7, 9, 22, 23) but also strengthens myeloid-derived suppressor cell (MDSC) activity (23), biases macrophages toward an M2 state *via* GPR132 (12), and supports Treg fitness under nutrient-poor conditions (10). Multiple strategies—oral buffering agents (24), biomaterial-based neutralizers (25, 26), proton pump inhibition (22), or metabolic interventions such as LDHA silencing (27) and MCT4 blockade (28)—can partially elevate tumor pH and improve immune competence. Many of these approaches enhance ICB efficacy (24, 27, 28) or promote adoptive immune cell therapy (22, 24, 29). However, systemically administered agents (24, 30, 31) often provide uneven intratumoral distribution, transient pH modulation, and limited control over the spatial gradient of acidity that shapes local immune function.

Electrochemical therapy (ECT), first introduced clinically by Nordenström in 1978 (32), uses inserted electrodes to drive *in situ* tumor electrolysis. Conventional ECT frequently employs inert electrodes (e.g., Pt/Pt), generating strong anodic acidification/oxidants and cathodic alkalinization (33) that can be cytotoxic but difficult to standardize and may cause nonspecific tissue damage (34–37). Importantly, because anodic and cathodic reactions are spatially separated, ECT also offers an underutilized opportunity to impose programmable physicochemical gradients *in vivo*. We reasoned that replacing the conventional inert anode with a sacrificial Fe anode could embed iron chemistry into ECT, enabling ferroptosis-associated killing while preserving the ability to modulate peritumoral pH.

We sought to leverage this principle through a spatially optimized, low-current ECT platform that separates tumor debulking from immune preservation. A central stainless-steel Fe anode [Fe(+)] is used to induce acidotoxic injury and initiate ferroptosis *via* Fe^2+^ release (38), while peripheral platinum (Pt) cathodes [Pt (–)] provide controlled alkalinization to counteract peritumoral acidosis—conditions that better support T-cell and macrophage function. Notably, ferroptosis can not only directly suppress tumor growth but can also induce immunogenic cell death and modulate the tumor immune microenvironment, thereby potentiating antitumor immune responses (39, 40). To amplify systemic antitumor immunity, we coupled this device with a plasmid-encoded αPD-1 single-chain antibody (scFv), produced in skeletal muscle (41) *via* a previously optimized electroporation system (42–46). This configuration yields sustained circulating PD-1 blockade while simultaneously reshaping the TME to be more permissive to immune attack.

We hypothesized that the coordinated actions of acidotoxic/ferroptotic tumor debulking (anode-driven) and relief of acidity-mediated immunosuppression (cathode-driven), together with sustained αPD-1 expression, would sensitize tumors to PD-1 blockade in a tumor-context-dependent manner. To test this, we used B16 melanoma as a mechanistic and safety platform to define a controllable electrochemical dosing window and to map intratumoral pH modulation, ferroptosis-associated killing, and immune microenvironment remodeling. We then evaluated whether the same ECT_Fe_ regimen could functionally sensitize an immune-refractory “cold” tumor, primarily in the 4T1-luc TNBC model.

## 2 Results

Overview: We first established and validated an optimized spatial Fe-anode/Pt-cathode electrochemical therapy (ECT_Fe_) platform. Because our primary therapeutic objective was to enhance the efficacy of PD-1 blockade in aggressive triple-negative breast cancer (TNBC), we evaluated the combination regimen primarily in the 4T1-luc TNBC model (Section 2.3). To define a controllable electrochemical dosing window and to derive mechanistic insights into how spatial electrochemistry shapes intratumoral pH, ferroptosis, and immune composition, we used B16 melanoma as a mechanistic and safety platform (Sections 2.1–2.2).

### 2.1 Optimized spatial Fe-anode/Pt-cathode ECT establishes a controllable pH gradient, therapeutic window, and antitumor efficacy in B16 melanoma

**Figure 1:**
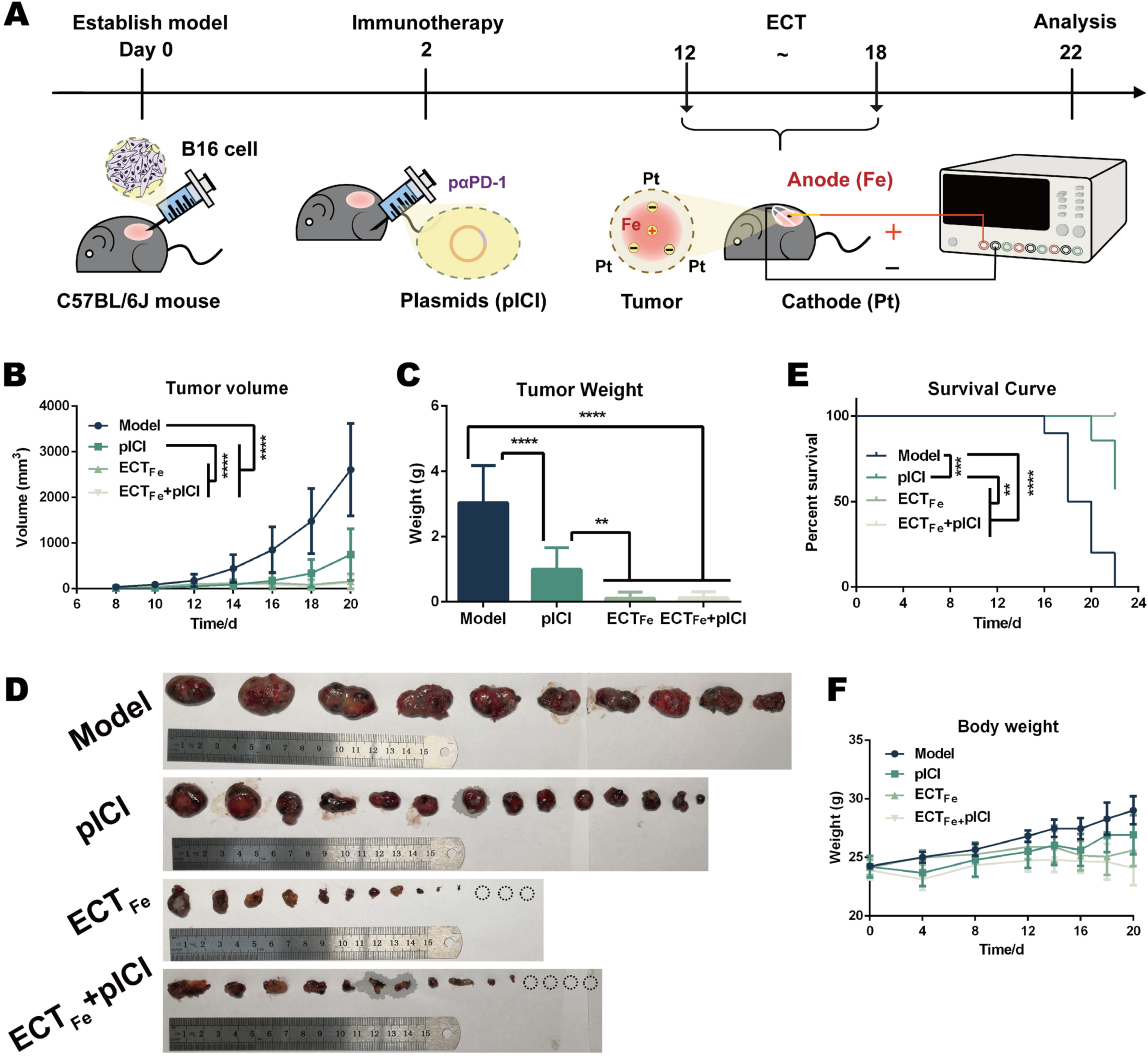
ECT_Fe_ achieves robust tumor control with minimal toxicity in B16 melanoma. **(A)** Treatment schematic. **(B)** Tumor growth curves. **(C)** Tumor weights at the end of treatment. **(D)** Excised tumors. **(E)** Kaplan–Meier survival curves. **(F)** Body weight. Data are presented as mean ± SD. Two-way ANOVA was used for tumor growth comparisons over time, one-way ANOVA for end-point tumor weight comparisons. Tumor efficacy data in **(B–F)** are from n ≥ 10 biologically independent mice per group. **p < 0.01; ***p < 0.001; ****p < 0.0001.

To validate our optimized Fe-anode/Pt-cathode spatial configuration *in vivo*, we first established a low-current ECT_Fe_ regimen in B16 melanoma to quantify spatial intratumoral pH dynamics and define a tolerable dosing window. With a centrally placed Fe anode and three peripheral Pt cathodes, ECT_Fe_ generated a steep but spatially confined pH gradient: rapid core acidification near the Fe anode (typically pH ~4.1–6.0 within the first few hours; **Supplementary Figure S1A**) and a transient, mild peritumoral pH elevation at the Pt sites (approximately pH ~6.6–7.4 over 4–24 h after treatment; **Supplementary Figure S1B**). Based on these measurements, we selected a safe and effective dosing window of 2 mA for 5 min per session, administered twice daily. Within this window, the anodal pH remained ~5.5–6.0 at 4 h, sufficient to induce acidotoxicity, whereas the cathodal pH remained ~6.6–7.0 at 4 h, consistent with moderate alkalinization and relief of local acidosis. This regimen was achieved without appreciable heating or procedural instability (**Supplementary Figures S1C–F**). Under these conditions, ECT_Fe_ produced robust tumor control and extended survival, outperforming PD-1 blockade alone in B16, whereas the ECT_Fe_+pICI group showed no additional benefit over ECT_Fe_ alone (**Figures 1A–E**). Throughout treatment, mice maintained stable body weight and showed no major abnormalities in blood chemistry/hematology or organ histology, supporting good tolerability of the optimized ECT_Fe_ regimen (**Figure 1F**; **Supplementary Figures S2A–M**).

### 2.2 *In vivo* mechanistic validation: ECT_Fe_ triggers ferroptosis-associated debulking and immune reconditioning in B16 tumors

**Figure 2:**
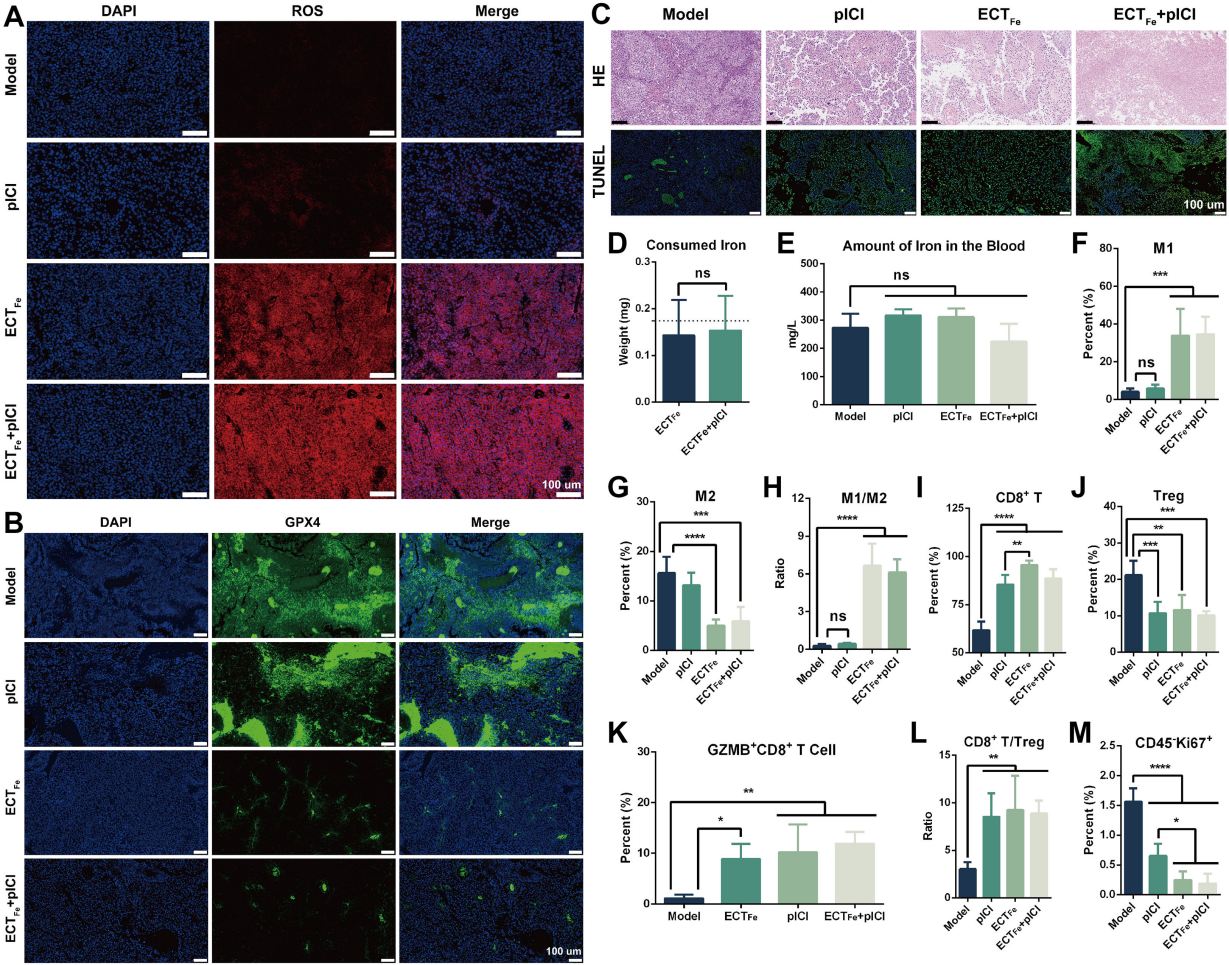
ECT_Fe_ induces ferroptosis-associated tumor cell death and immune reconditioning in B16 melanoma. **(A)** Representative fluorescence images of ROS in frozen tumor sections. **(B)** GPX4 expression in tumor tissue assessed by immunofluorescence, showing reduced GPX4 signal. **(C)** Representative H&E staining and TUNEL assay of tumor sections, highlighting treatment-induced cell death. **(D)** Quantification of iron anode mass loss after ECT_Fe_ treatment (dashed line: theoretical loss; n ≥ 14). **(E)** Serum iron concentration (n = 3 mice per group), reflecting iron release from the Fe anode. **(F–H)** Flow cytometric analysis of tumor-associated macrophage polarization, showing the proportions of M1 (pro-inflammatory) and M2 (immunosuppressive) macrophages. **(I–L)** Flow cytometric quantification of tumor-infiltrating immune cells after treatment, including CD8^+^ T cells **(I)**, Tregs **(J)**, activated CD8^+^ T cells **(K)**, and the CD8^+^ T cell to Treg ratio **(L)**. **(M)** Flow cytometry measurement of tumor cell proliferation (percentage of Ki67^+^ cells among CD45^-^ tumor cells) in treated *vs*. model tumors. Flow cytometry analysis was performed with n = 5 biologically independent mice per group **(F–M)**. Data are presented as mean ± SD. One-way ANOVA was used for multi-group comparisons. Images in **(A–C)** are representative of n = 3 independent tumors per group. ns, not significant; *p < 0.05; **p < 0.01; ***p < 0.001; ****p < 0.0001. Scale bar, 100 μm.

Having defined a safe, controllable dosing window, we next interrogated how ECT_Fe_ drives tumor debulking and microenvironment remodeling *in vivo*. Consistent with the Fe-anode chemistry, treated tumors displayed widespread oxidative stress (ROS staining) and marked depletion of the lipid peroxide detoxifying enzyme GPX4, together with extensive histological damage and TUNEL-positive cell death (**Figures 2A–C**). Quantification of anode consumption closely matched Faraday’s-law predictions, while serum iron remained unchanged, indicating that Fe release was largely confined to the tumor site rather than systemically distributed (**Figures 2D, E**).

Beyond direct cytotoxicity, ECT_Fe_ induced systemic and intratumoral immunologic changes consistent with microenvironment reconditioning. Serum cytokines shifted toward a proinflammatory profile (increased IFN-γ, TNF-α, IL-6 and decreased IL-10) (**Supplementary Figures S3A–D**). Flow cytometry revealed increased M1-like macrophages (CD86^+^) and reduced M2-like macrophages (CD206^+^), leading to a higher M1/M2 ratio (**Figures 2F–H**; **Supplementary Figure S3E**). Concomitantly, CD8^+^ T-cell infiltration and activation (granzyme B) increased, Tregs decreased, and the CD8^+^/Treg ratio improved, accompanied by reduced tumor-cell proliferation (Ki-67) (**Figures 2I–M**; **Supplementary Figures S3F, G**). Together, these data indicate that spatial electrochemistry can couple ferroptosis-associated debulking with relief of acidity-driven immunosuppression, motivating us to test whether ECT_Fe_ could translate into functional sensitization of PD-1 blockade in the immune-refractory 4T1-luc TNBC model (Section 2.3).

### 2.3 ECT_Fe_ sensitizes 4T1-luc triple-negative breast cancer to PD-1 blockade

**Figure 3:**
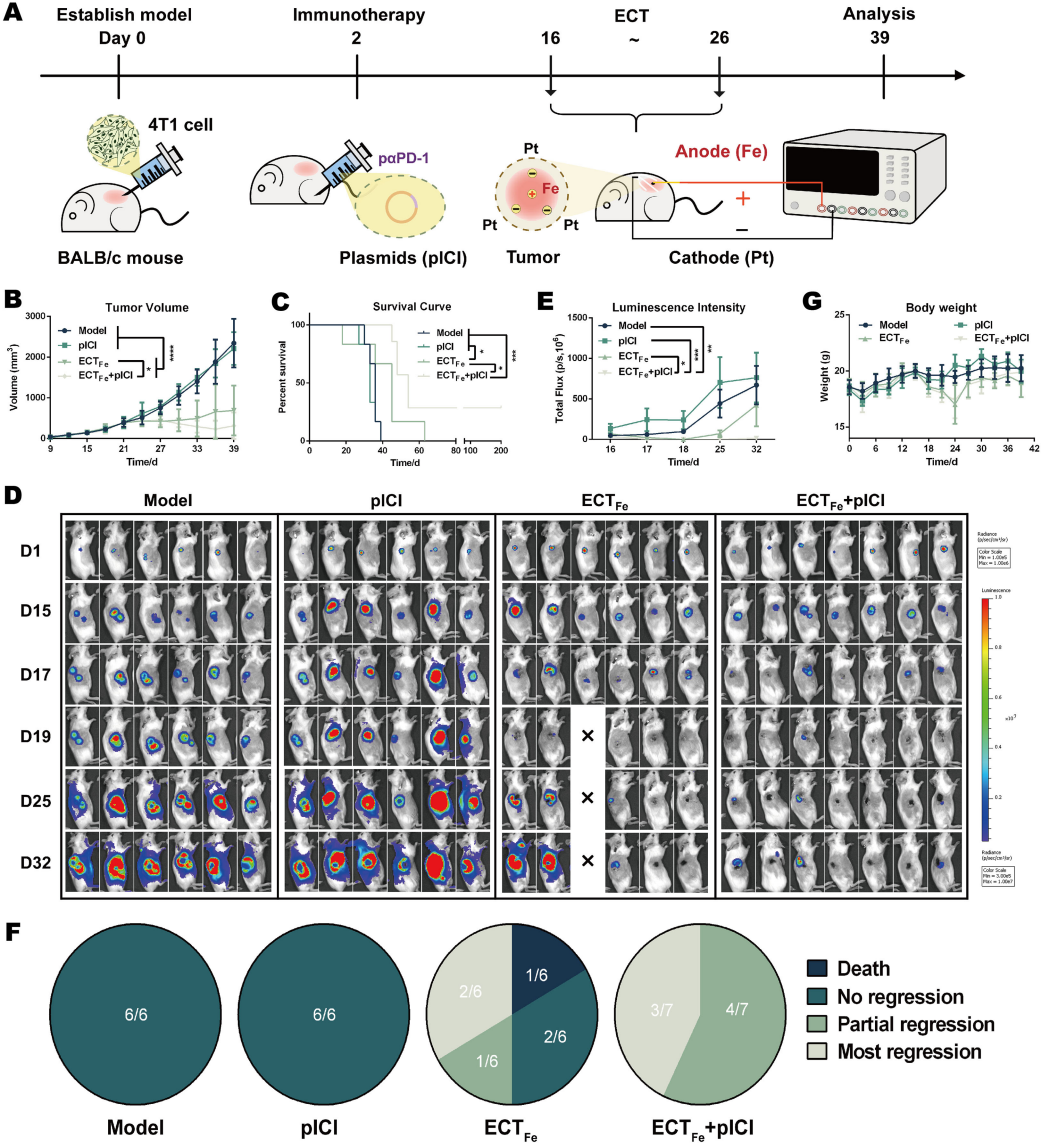
ECT_Fe_ sensitizes 4T1-luc TNBC to PD-1 blockade and drives tumor regression. **(A)** Timeline of treatment. **(B)** Tumor volume. **(C)** Kaplan–Meier survival curves of the mice. **(D)** Bioluminescence images of the mice. **(E)** Quantification of tumor bioluminescence signal. **(F)** Tumor status on day 32 assessed from bioluminescence imaging. **(G)** Body weight of the mice during therapy, indicating tolerability. Data are presented as mean ± SD. Two-way ANOVA was used for tumor volume comparisons over time, and survival was analyzed by log-rank test. n ≥ 6 biologically independent mice per group **(B–G)**. ns, not significant; *p < 0.05; **p < 0.01; ***p < 0.001; ****p < 0.0001.

We next tested whether the immune-permissive changes induced by ECT_Fe_ translate into enhanced checkpoint efficacy in an aggressive TNBC setting. Using luciferase-expressing 4T1 cells (4T1-luc), tumor burden was monitored noninvasively by bioluminescence (**Supplementary Figure S4**). In contrast to the B16 melanoma model, where PD-1 blockade added little incremental benefit to ECT_Fe_, the 4T1-luc TNBC model showed a clear combination advantage: ECT_Fe_ plus pICI outperformed either monotherapy, slowing tumor growth and inducing regression (**Figures 3A, B**). Tumors in the combination group gradually regressed from day 21 to 36, whereas tumors in control or monotherapy groups progressed. Accordingly, combination therapy significantly prolonged survival (**Figure 3C**).

Bioluminescence imaging corroborated tumor regression: combination-treated mice displayed minimal luminescent signal at the primary site and no obvious distant metastases, whereas controls retained strong signals (**Figures 3D, E**). Several mice achieved complete regression with no palpable tumor (**Figure 3F**). A transient body-weight decrease occurred during ECT but recovered after treatment, indicating tolerability (**Figure 3G**).

### 2.4 Mechanistic insights into ECT_Fe_-induced cytotoxicity *in vitro*

**Figure 4:**
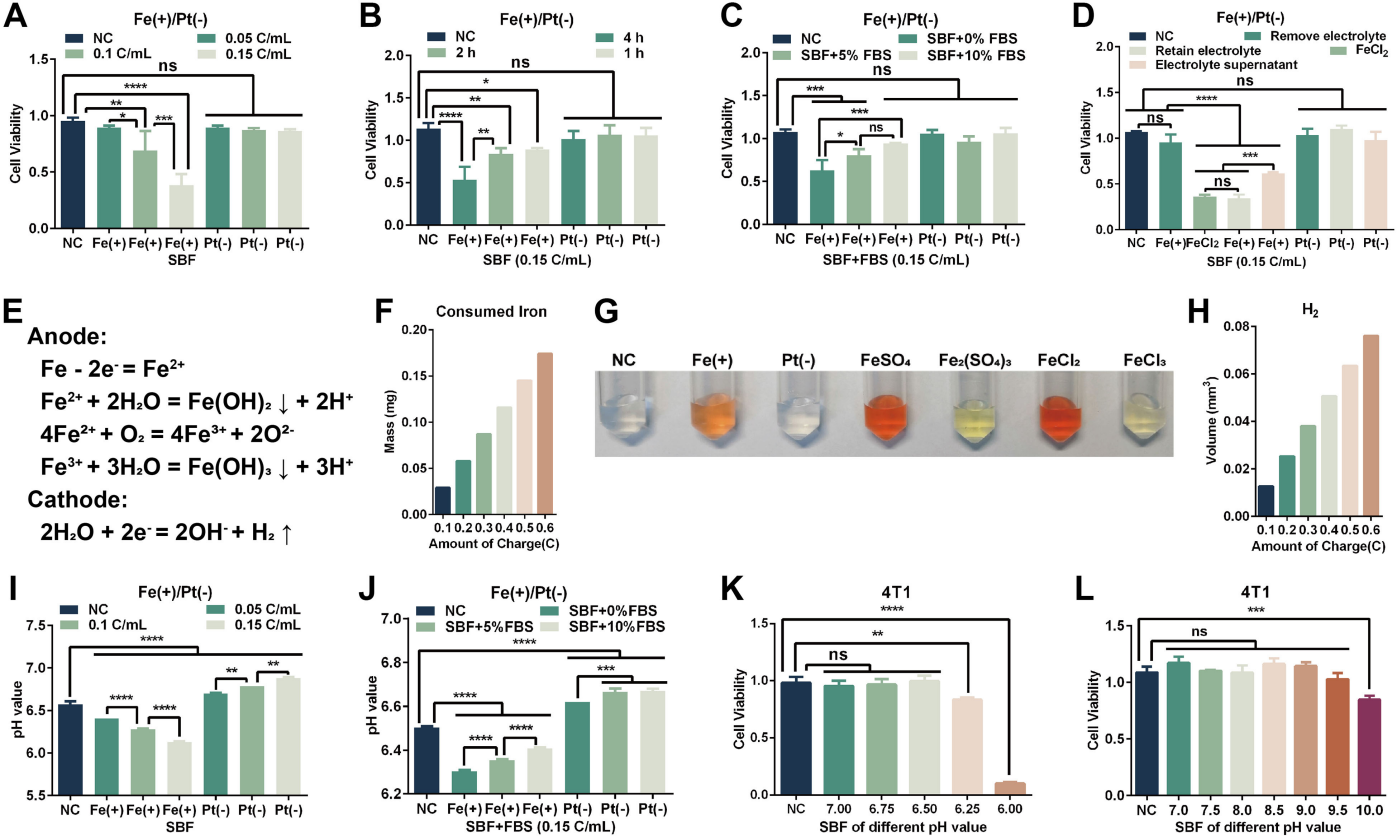
*In vitro* cytotoxicity and electrochemical effects of ECT_Fe_ on 4T1 cells. **(A, B)** Anodic cytotoxicity is dependent on the charge density and electrolyte exposure time, while cathodic cytotoxicity is negligible. Cell viability in SBF after a 4-h incubation with varying total charge densities (C/mL) **(A)**, or at a fixed charge density of 0.15 C/mL across different duration (1–4 h) **(B)**. **(C)** Effect of serum proteins on cytotoxicity: cell viability after a 4-h ECT_Fe_ electrolyte treatment in SBF with graded FBS concentrations, demonstrating that FBS attenuates cytotoxicity. **(D)** Cell viability under different exposure conditions: continuous 4-h exposure to freshly electrolyzed SBF; immediate removal of electrolyte followed with a 4-h incubation in normal cell culture medium; and exposure to cell-free electrolyzed SBF supernatant for 4 h. **(E)** Schematic of the electrochemical reactions at the electrodes during ECT_Fe_ treatment (iron oxidation at the anode and water reduction with H_2_ gas evolution at the cathode). **(F)** Theoretical mass of iron dissolved from the anode as a function of electric charge (Faraday’s law calculation). **(G)** Detection of Fe^2+^ released into the electrolyte during Fe-anode electrolysis using an o-phenanthroline colorimetric assay. **(H)** Theoretical volume of H_2_ gas generated at the cathode as a function of charge (calculated using Faraday’s law and the ideal gas equation). **(I, J)** Dose-dependent pH changes after ECT_Fe_ electrolysis in pure SBF **(I)** and in SBF containing FBS **(J)**, showing the buffering effect of FBS on pH. **(K, L)** Cell viability after 4-h exposure to SBF adjusted to an acidic pH **(K)** or an alkaline pH **(L)**, mimicking the anodic and cathodic pH conditions, respectively. Data are presented as mean ± SD. n = 3 independent experiments. One-way ANOVA was used for comparisons among groups. ns, not significant; *p < 0.05; **p < 0.01; ***p < 0.001; ****p < 0.0001.

To dissect the direct tumoricidal effects of ECT_Fe_, we performed *in vitro* studies with 4T1 cells under various electrochemical conditions. The cytotoxicity of ECT_Fe_ depended strongly on the total electric charge delivered and the electrolyte exposure duration. In the simulated body fluid (SBF), increasing the charge density applied *via* the Fe anode led to progressively lower 4T1 cell viability, with maximal cell death at the highest charge tested (**Figure 4A**). Similarly, prolonged electrolyte exposure (1–4 h) under a fixed current dose caused a time-dependent decrease in viability (**Figure 4B**). These results indicate that the extent of cell killing is directly linked to the electrochemical dose delivered and the duration of exposure. Notably, the Pt cathode alone had minimal impact on cells, confirming that the primary cytotoxic effects stem from the Fe anode’s activity. The composition of the surrounding medium also significantly influenced outcomes: the presence of serum proteins attenuated the cytotoxicity of ECT_Fe_. When treatments were carried out in buffer supplemented with increasing concentrations of fetal bovine serum (FBS), cell survival improved in a dose-dependent manner (**Figure 4C**). Flow cytometry quantification (**Supplementary Figure S5A**) and Live/dead fluorescence staining (**Supplementary Figure S5B**) confirmed this protective effect of serum. Moreover, conducting electrolysis in complete cell culture medium (which contains 10% FBS, providing buffering agents and antioxidants) markedly reduced cytotoxicity (**Supplementary Figure S5C**), suggesting that medium components can neutralize the toxic reactive species generated during electrolysis.

We next examined how continuous exposure contributes to cell death. Cells continuously exposed to the electrolyte in SBF for 4 hours after electrolysis suffered extensive killing. In contrast, cells that were only briefly exposed—with the electrolyzed solution immediately removed and replaced with fresh cell culture medium post−electrolysis—showed significantly higher survival compared to continuous exposure (**Figure 4D**). Likewise, exposing cells to the cell-free electrolyzed SBF supernatant for 4 h produced intermediate toxicity (**Figure 4D**). These observations indicate that the reactive species generated by electrolysis, which reside in the electrolyzed solution, are the primary source of cytotoxicity.

To further characterize the electrochemical properties of the electrolyte, we measured the species generated during ECT_Fe_. Theoretical calculations based on known electrochemical reactions (**Figure 4E**; **Supplementary Figure S6A**) indicated that the amount of ferrous iron (Fe^2+^) dissolved from the anode is proportional to the total charge passed (Faraday’s law; **Figure 4F**). We qualitatively confirmed the presence of Fe^2+^ in the electrolyte using an *o*-phenanthroline colorimetric assay, which showed an orange color indicative of Fe^2+^ (**Figure 4G**). Concurrently, water reduction at the Pt cathode was predicted to generate hydrogen gas in proportion to charge (**Figure 4H**), according to Faraday’s law and the ideal gas equation (**Supplementary Figure S6A**). Based on the electrochemical reaction equations (**Figure 4E**), the hydrolysis of Fe^2+^ at the anode generates H^+^, leading to local acidification. Meanwhile, the hydrogen evolution reaction (HER) at the cathode produces OH^−^, resulting in local alkalinization. Direct pH measurements revealed significant local pH shifts during electrolysis: in serum-free buffer, the anodic region’s pH dropped sharply while the cathodic region’s pH rose (**Figure 4I**). The inclusion of serum mitigated these extremes by buffering the pH toward physiological levels (**Figure 4J**; **Supplementary Figure S6B**). Indeed, pH changes emerged as a major factor in cytotoxicity: direct exposure of cells to an acidic pH equivalent to that produced near the Fe anode drastically reduced viability (**Figure 4K**), whereas exposure to an alkaline pH comparable to the Pt cathode environment had little effect on cell survival (**Figure 4L**). Consistently, fluorescence imaging confirmed extensive cell death and elevated ROS in 4T1 cells subjected to low pH alone (**Supplementary Figures S6C, D**). Thus, the acidification caused by the Fe anode reaction is a key driver of cell injury, while the moderate alkalinization at the cathode is safe and well tolerated.

We found that ECT_Fe_ did not directly generate certain reactive oxygen species during electrolysis. Methylene blue (MB) indicator assays showed no detectable hydroxyl radicals (•OH) at the anode but did detect molecular H_2_ production at the cathode (**Supplementary Figures 7SA–C**). In addition, during *in vitro* ECT_Fe_ (**Supplementary Figure S7D**), the voltage progressively declined (**Supplementary Figure S7E**). Post-electrolysis, a precipitate formed in the anode solution, contrasting with the clear cathode solution (**Supplementary Figure S7F**), and visible etching was observed on the stainless steel needle (**Supplementary Figure S7G**). Taken together, these *in vitro* results show that ECT_Fe_’s cytotoxic effect is governed by electrochemical parameters – charge dosage and exposure duration – and is modulated by the composition of the surrounding medium. Acidic corrosion products and iron-derived species are identified as the primary mediators of tumor cell death under *in vitro* ECT_Fe_ conditions.

### 2.5 Fe anode induces ferroptotic cell death in 4T1 cells *via* iron overload and lipid peroxidation

**Figure 5:**
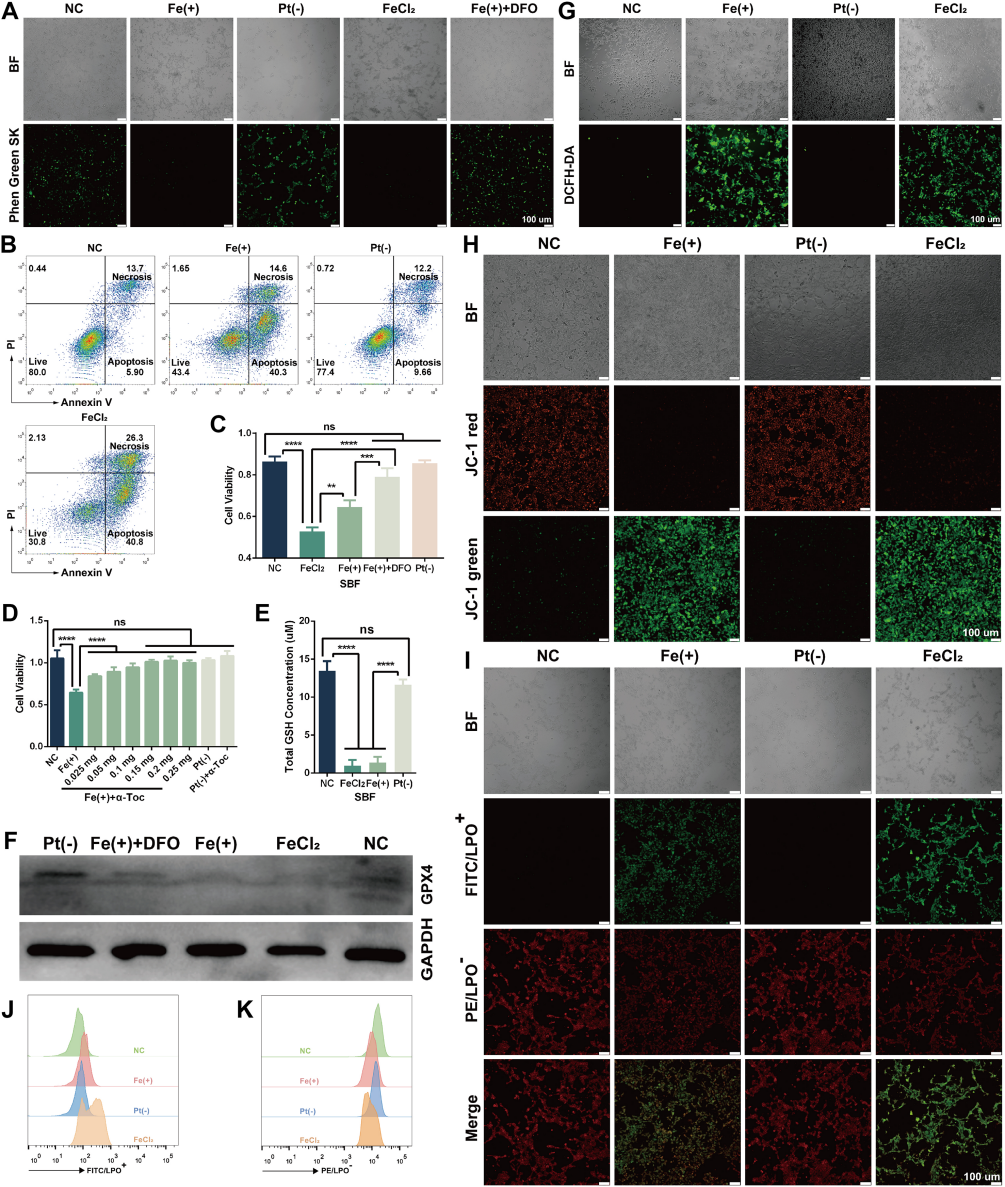
Fe-anode induces ferroptosis of 4T1 cells in ECT_Fe_ *in vitro***. (A)** Intracellular free iron accumulation in 4T1 cells after treatment, as shown by fluorescence microscopy using Phen Green SK probe (green fluorescence is quenched by Fe^2+^). **(B)** Overall cell death quantified by flow cytometry after electrolysis. **(C, D)** Rescue of cells by ferroptosis inhibitors: co-treatment with DFO (iron chelator) or α-Toc (lipid peroxidation inhibitor) significantly protects 4T1 cells from ECT_Fe_-induced death. **(E)** Intracellular GSH levels after treatment, showing significant GSH depletion consistent with ferroptosis. **(F)** GPX4 protein levels showed a marked reduction after ECT_Fe_ treatment, which was prevented by DFO co-treatment. **(G)** Detection of elevated intracellular ROS using the DCFH-DA fluorescence probe after treatment. **(H)** Loss of mitochondrial membrane potential after treatment was visualized by JC-1 staining (red: J-aggregates; green: monomers). **(I)** Fluorescence microscopy images of lipid peroxidation (LPO) in 4T1 cells stained with the Lipoxite R590/G525 sensor, showing increased LPO (oxidation causes a shift from red to green fluorescence) after treatment. **(J, K)** Flow cytometry analysis of LPO. Data are presented as mean ± SD. n = 3 independent experiments. One-way ANOVA was used for comparisons among groups. ns, not significant; *p < 0.05; **p < 0.01; ***p < 0.001; ****p < 0.0001. Scale bar, 100 μm.

We then investigated whether the cell death induced by the Fe anode is ferroptosis – an iron-dependent, lipid peroxidation–driven form of cell death. 4T1 cells exposed to ECT_Fe_ electrolyte *in vitro* displayed multiple hallmarks of ferroptosis. Intracellular labile iron accumulated substantially in electrolyte-treated cells, demonstrated by quenching of the iron-sensitive dye Phen Green SK (loss of green fluorescence indicates Fe^2+^ binding; **Figure 5A**). This iron overload was accompanied by widespread cell death (**Figure 5B**) and a surge in oxidative stress. To directly test ferroptosis involvement, we assessed the effect of known ferroptosis inhibitors on cell survival. Co-treatment with the iron chelator deferoxamine (DFO) or the lipophilic antioxidant α-tocopherol (α-Toc) provided significant protection against ECT_Fe_-induced death, rescuing a large fraction of 4T1 cells (**Figures 5C, D**). We also measured biochemical markers of ferroptosis in treated cells. Intracellular glutathione (GSH) levels were drastically depleted after ECT_Fe_ treatment (**Figure 5E**), consistent with unchecked lipid peroxidation consuming the cell’s antioxidant reserves. Correspondingly, the expression of GPX4 – an essential enzyme that detoxifies lipid peroxides – was dramatically reduced in 4T1 cells following Fe anodic electrolyte exposure (**Figure 5F**). Notably, GPX4 loss was prevented when excess iron was chelated by DFO (**Figure 5F**), linking iron overload to GPX4 inactivation. These results establish that Fe-based ECT kills cancer cells by inducing ferroptosis, driven by iron overload and lipid peroxidation.

Electrolyte-treated cells also exhibited markedly elevated ROS levels (**Figure 5G**) and a collapse of mitochondrial membrane potential (loss of red JC-1 aggregates, **Figure 5H**), consistent with extensive oxidative damage. Crucially, we observed a significant increase in lipid peroxidation in ECT_Fe_-treated cells. Fluorescent lipid peroxidation sensors showed a pronounced shift from red to green fluorescence, indicating high lipid peroxide content in treated cells (**Figure 5I**). Flow cytometry further verified this increase in lipid peroxidation products (**Figures 5J, K**). These findings indicate that Fe anodic electrolyte exposure creates an environment of iron excess and oxidative stress that drives lethal lipid peroxidation in 4T1 cells. The combination of iron accumulation, GSH depletion, GPX4 downregulation, and rescue by ferroptosis inhibitors provides compelling evidence that ferroptotic cell death is the predominant mechanism underlying the Fe anode’s cytotoxicity in ECT_Fe_.

### 2.6 Acidic pH suppresses while neutral-to-alkaline pH promotes immune cell function

**Figure 6:**
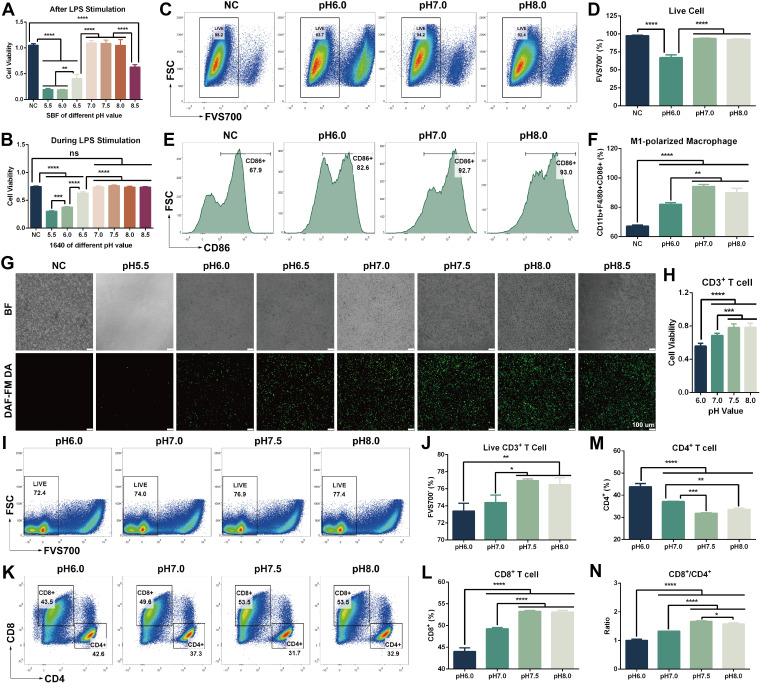
pH effects on the viability and function of immune cells *in vitro*. **(A)** Following a 24-h LPS priming (activation), the viability of RAW 264.7 macrophages were measured after a 4-h exposure to SBF at indicated pH. **(B)** Viability of RAW 264.7 after 24 h incubation in complete culture medium (with LPS) at indicated pH. **(C, D)** Flow cytometry quantification of live RAW 264.7 cells after 24 h LPS stimulation under various pH conditions. **(E, F)** Flow cytometry analysis of macrophage polarization under different pH conditions (percentage of cells exhibiting an M1 phenotype after LPS stimulation at each pH). **(G)** Fluorescence imaging of intracellular NO production in M1-polarized RAW 264.7 cells after 24 h at various pH, using the DAF-FM DA probe (green fluorescence indicates NO; images show representative M1 cells at alkaline/neutral *vs*. acidic pH). **(H)** Viability of primary CD3^+^ T cells after a 24-h culture in T-cell medium adjusted to the indicated pH. **(I, J)** Flow cytometric analysis of CD3^+^ T cell viability after 24 h culture at different pH. **(K–N)** Analysis of CD4^+^ and CD8^+^ T cell subsets within the CD3^+^ T cell population after 24 h at different pH conditions. Data are presented as mean ± SD. n = 3 independent experiments. One-way ANOVA was used to compare groups at different pH. ns, not significant; *p < 0.05; **p < 0.01; ***p < 0.001; ****p < 0.0001. Scale bar, 100 μm.

The functionality of immune cells was found to be highly pH-dependent *in vitro* (**Figure 6**). Macrophages: M1-polarized macrophages were sensitive to severe acidosis (pH 5.5–6.5) but survived normally at neutral-to-alkaline pH (7.0–8.0), with only a significant decline at the highest basic pH (8.5) (**Figure 6A**). Consistently, polarization to the M1 phenotype was more efficient in neutral or alkaline environments. Cultures maintained at pH 7.0–8.5 yielded higher cell numbers and viability than those at acidic pH 5.5–6.5 (**Figure 6B**). Flow cytometry confirmed this trend: the fraction of live macrophages was significantly higher under neutral/alkaline conditions and significantly lower under acidic conditions (**Figures 6C, D**). Meanwhile, the frequency of M1-polarized macrophages was markedly higher under neutral/alkaline conditions than under acidic conditions, indicating enhanced proinflammatory polarization at higher pH (**Figures 6E, F**). Furthermore, nitric oxide (NO) production – a hallmark of M1 activation – was negligible at pH 5.5, increased at pH 6.0–6.5, and peaked at pH 7.0–8.0, paralleling the pattern of M1 cell viability and differentiation (**Figure 6G**). Collectively, these findings demonstrate that a neutral-to-alkaline pH supports overall macrophage function by enhancing both viability and proinflammatory polarization, in contrast to the suppressive effects of acidosis.

T cells: T lymphocyte survival and subset balance were also modulated by pH (**Figures 6H–N**). T cell viability was significantly higher at neutral-to-alkaline pH (7.0–8.0) compared to acidic pH (6.0) (**Figure 6H**). Flow cytometry corroborated these results, showing a much greater proportion of live T cells at pH 7.0–8.0 than at pH 6.0 (**Figures 6I, J**). Moreover, neutral/alkaline pH shifted the composition of T cell subsets in a way that favors anti-tumor immunity: the percentage of CD8^+^ T cells increased markedly with higher pH, while CD4^+^ T cells decreased (**Figures 6K–M**). This led to a significantly elevated CD8^+^/CD4^+^ ratio under neutral and basic conditions relative to acidic culture (**Figure 6N**). In summary, acidic conditions (pH 6.0) impaired overall T cell survival and skewed the repertoire towards CD4^+^ cells, whereas neutral/alkaline conditions (pH 7.0–8.0) preferentially supported the survival of cytotoxic CD8^+^ T cells.

Collectively, these *in vitro* results underscore that acidosis broadly disrupts critical immune functions, whereas a neutral to slightly alkaline microenvironment supports macrophage M1 polarization and T cell viability – preferentially expanding the cytotoxic CD8^+^ T cell subset – to potentially promote a more robust antitumor immune response.

## 3 Discussion

Research Question and Main Conclusions: Our central goal was to develop an optimized spatial electrochemical therapy (ECT_Fe_; Fe(+)/Pt (–) with a central Fe anode and peripheral Pt cathodes) that reconditions the acidic tumor microenvironment (TME) to sensitize immune-checkpoint-refractory triple-negative breast cancer (TNBC) to PD-1 blockade. In the 4T1-luc TNBC model, ECT_Fe_ combined with intramuscularly expressed αPD-1 scFv (pICI) produced tumor regression and significantly prolonged survival compared with either monotherapy (**Figure 3**). By contrast, in B16 melanoma, ECT_Fe_ alone achieved strong tumor control and survival benefit and exceeded PD-1 blockade alone, while adding PD-1 blockade provided little additional gain (**Figure 1**), underscoring that combination benefit is tumor-context dependent. We speculate that this limited incremental gain in melanoma may reflect its relatively immunogenic “hot” phenotype and/or the strong standalone efficacy of ECT_Fe_ in that setting. Using B16 as a mechanistic platform, we mapped how the Fe anode creates an acidic, Fe^2+^-rich core with ROS elevation and GPX4 suppression consistent with ferroptosis (**Figure 2**), while peripheral Pt cathodes provide mild pH relief that supports effector immune function and drives a more proinflammatory macrophage/T-cell landscape (**Figures 1**, **2**).

Key Innovations and Comparison to Prior Work: 1) Optimized Fe(+)/Pt (–) spatial programming to embed ferroptosis into ECT. Unlike conventional inert Pt(+)/Pt (–) electrolysis that relies primarily on acid/base extremes and oxidants, a sacrificial Fe anode introduces localized iron chemistry and ferroptosis-associated killing while preserving spatial pH control. 2) Microenvironmental engineering for ICB sensitization, particularly in “cold” tumors. Compared with systemic buffers or metabolic interventions that can yield heterogeneous and transient pH modulation, electrode geometry provides immediate, spatially constrained pH relief at the tumor margin where immune cells operate. 3) Practical checkpoint delivery. Muscle-based expression of plasmid-encoded αPD-1 scFv enables sustained systemic PD-1 blockade without repeated antibody dosing.

Collectively, these features support an electro-immunotherapy paradigm in which localized ferroptotic/acidotoxic tumor debulking and peritumoral pH relief cooperate to make the TME more compatible with checkpoint activity. Notably, the magnitude of benefit from adding PD-1 blockade is tumor-type dependent, with the strongest combination advantage observed here in immune-refractory TNBC.

Limitations: First, ECT_Fe_ requires invasive electrode placement, which may limit application to deep-seated or multifocal disease and may introduce variability due to tumor size and conductivity. Second, the benefit of adding PD-1 blockade is context dependent; identifying predictive tumor features (e.g., baseline acidity, immune exclusion/”cold” status, or ferroptosis susceptibility) will be important for translation. Third, the *in vivo* spatiotemporal distribution of reactive species and iron chemistry remains incompletely resolved, and physiological buffering/antioxidant systems may attenuate ferroptotic intensity, requiring careful dose calibration across tumor types.

Unresolved Issues and Possible Solutions: Key questions include whether ECT_Fe_ can elicit abscopal effects and durable immunological memory, and how electrode geometry and dosing should be individualized to tumor size and heterogeneity. Closed-loop control using real-time readouts (e.g., pH, impedance, or electrode potential) could help standardize delivery and avoid over- or under-treatment. From an immunology standpoint, testing additional combinations (e.g., STING/TLR agonists, co-stimulatory agonists, or dual-checkpoint blockade) may clarify how broadly this microenvironmental engineering strategy can convert non-responsive tumors into ICB-responsive disease.

Insights and Future Directions: Our results support a model in which Fe-anode-driven ferroptotic/acidotoxic tumor debulking provides inflammatory cues, while Pt-cathode-mediated pH relief preserves local effector function, together priming tumors for PD-1 blockade. In TNBC, where checkpoint response is often limited, this approach yielded a pronounced combination benefit. Future work should (i) define biomarkers that predict when ECT_Fe_ will sensitize PD-1 blockade, (ii) quantify distal immune effects in metastatic and rechallenge settings, (iii) test additional poorly immunogenic tumor models, and (iv) advance minimally invasive, image-guided electrode deployment and standardized dosing for clinical translation.

## 4 Materials and methods

### 4.1 Reagents and materials

Dimethyl sulfoxide (DMSO) was purchased from Solarbio. α-Tocopherol (α-Toc) was from Energy Chemical, deferoxamine (DFO) and methylene blue (MB) from Macklin. Simulated body fluid (SBF; with or without chloride) was from Yuan Ye Biotechnology. o-Phenanthroline was from Kemiou, and Phen Green SK from GLPBIO. DAF-FM DA and lipopolysaccharide (LPS) were from Beyotime. Hematoxylin and eosin (H&E) staining kits and TUNEL apoptosis detection kits were from Li Lai Biotechnology. The Cell Counting Kit-8 (CCK-8) was purchased from Abbkine. An infrared forehead thermometer was from CEM (Shenzhen, China).

### 4.2 Plasmid construction and preparation

A plasmid encoding mouse anti-PD-1 scFv (αPD-1, referred to as pICI) was constructed following established protocols (41). The variable region of the PD-1 antibody was based on patent EP1445264A1, and the sequence was inserted into the pEMS plasmid containing a muscle-specific EMS promoter (Patent CN113106094B). Plasmids were purified using an EndoFree Plasmid Kit (Cwbio, Jiangsu, China), and concentrations were determined with a NanoDrop 2000 spectrophotometer.

### 4.3 Cell lines and culture

The HEK293T (RRID: CVCL_0063), B16 (RRID: CVCL_F936), RAW264.7 (RRID: CVCL_0493), and 4T1 (RRID: CVCL_0125) cell lines were obtained from the Cell Bank of the Chinese Academy of Sciences (Shanghai, China) in October 2018, October 2018, November 2020, and June 2021, respectively. Upon receipt, all cells were expanded and frozen down at low passage. According to the cell bank’s quality control report, the cell lines were tested and confirmed to be free of mycoplasma contamination, and their identities were authenticated by short tandem repeat (STR) profiling. HEK293T and RAW264.7 cells were maintained in high-glucose DMEM + 10% FBS + 1% penicillin–streptomycin (P/S). 4T1 and B16 cells were maintained in RPMI-1640 + 10% FBS + 1% P/S. A luciferase-expressing 4T1 line (4T1-luc) was established *via* lentiviral transduction and selection with 2 μg/mL puromycin. T cells were cultured in RPMI-1640 with 10% FBS, 1% P/S, 0.05 mM β-mercaptoethanol, 0.1 mM NEAA, 1 mM sodium pyruvate, 2 mM L-glutamine, and 50 U/mL IL-2. All cells were maintained in a 37 °C, 5% CO_2_ incubator.

### 4.4 *In vitro* electrochemical treatments

4T1 cells were seeded in 24-well plates and grown for 24 h. After PBS washing, 2 mL SBF (pH 6.5) was added per well. Stainless steel (0.35 mm diameter) and platinum (0.3 mm diameter) electrodes, arranged as Fe anode/Pt cathode, were suspended across adjacent wells connected by a filter paper bridge to avoid direct contact with cells. A DC current was applied using a KA3303P or HSPY-500–02 source. Post-electrolysis, the electrolytes were either immediately removed or left in place for further incubation (typically 4 h for ECT_Fe_) before assessing cell viability or death mechanisms.

### 4.5 *In vivo* tumor models and treatments

Subcutaneous tumor models were established by injecting 1.5×10^5^ B16 melanoma cells in C57BL/6J mice or 1.25×10^5^ 4T1 breast cancer cells in BALB/c mice. On day 2 post-inoculation, immunotherapy was administered *via* intramuscular delivery of 100 μg of pαPD-1 plasmid into the bilateral tibialis anterior (TA) muscles of each mouse using L64-assisted electroporation (L/E/G system) (42–44). ECT was initiated when tumors reached 100–200 mm³, using Fe anode/Pt cathode electrode configurations powered by a KA3303P supply. For ECT, one anode (Fe) was inserted centrally into the tumor and three Pt cathodes placed around the tumor periphery (3–5 mm from the anode). Electrode depth was adjusted according to tumor thickness to ensure that the needles remained within the tumor without perforating normal tissue; the maximum insertion depth was 0.5 cm to minimize damage to surrounding healthy tissue. A constant current of 2 mA was applied per electrode set, delivered twice in one day (with a 6-h interval). B16 tumors received ECT for 1 day (5 min per session), while 4T1-luc tumors received ECT on 3 consecutive days (5 min per session). During the second or subsequent ECT sessions, the cathodes were inserted at positions different from those used in each previous session to expand the area of cathodal alkalinization and avoid cumulative damage resulting from repeated alkalinization effects. Tumor volumes were measured with calipers and calculated as (length × width²)/2. Mice were sacrificed when the tumor volume reached 4,000 mm^3^.

### 4.6 Flow cytometry

Tumors were harvested (day 19 for B16 experiment) and dissociated in collagenase I (100 U/mL), collagenase IV (400 U/mL), and DNase I (30 U/mL) for 30 min at 37 °C. Single-cell suspensions were treated with RBC lysis buffer and Fc-block (anti-CD16/32), then stained with Fixable Viability Stain 700 (BD) to exclude dead cells. For tumor cells, Ki-67 was stained intracellularly (anti-Ki-67-BV605). For infiltrating immune cells, panels included: T cells – CD45-V500, CD3-FITC, CD4-APC, CD8-PerCP-Cy5.5, GZMB-Alexa750; Tregs – CD45, CD4, CD25-BV421, Foxp3-PE; Macrophages – CD45, CD11b-PerCP-Cy5.5, F4/80-PE, CD86-PE-Cy7 (M1), CD206-AF647 (M2). After surface and intracellular staining (Foxp3/Transcription Factor Staining Buffer Set, eBioscience), data were acquired on a BD LSRFortessa and analyzed with FlowJo.

For *in vitro* analyses, 4T1 cells were treated with electrolyzed SBF as described and stained using: Live/Dead stain (Beyotime), Annexin V/PI apoptosis kit (Beyotime), or a lipid peroxidation sensor (Lipoxite R590/G525, AAT Bioquest), then analyzed by flow cytometry. For macrophage polarization *in vitro*, RAW264.7 cells were cultured for 24 h in LPS (100 ng/mL)-supplemented media adjusted to various pH, then stained for viability (FVS700) and markers (CD11b, F4/80, and CD86). For T cell activation *in vitro*, splenic CD3^+^ T cells were isolated (EasySep Mouse T Cell Isolation Kit, STEMCELL) and cultured in media at different pH for 24 h, then stained (FVS700, CD3, CD4, CD8) to assess viability and subset phenotypes.

### 4.7 pH measurements

Electrolyte pH was measured with a LE422 microprobe (Mettler-Toledo) after two-point calibration. Intratumoral pH after ECT was measured with needle microelectrodes (MI-407 pH electrode with MI-402 reference electrode, Microelectrodes Inc.) and a Delta 320 pH meter (Mettler-Toledo).

### 4.8 Hydroxyl radical and hydrogen detection

Hydroxyl radicals (•OH) and hydrogen (H_2_) generation during electrolysis were monitored *via* a MB decolorization assay. MB (0.2 mg/mL) was added to collected anode and cathode supernatants; absorbance at 664 nm was recorded over 180 min. MB degradation reflects oxidizing (•OH) or reducing (H_2_) species production: oxidation causes MB bleaching (lower absorbance), while reduction can also consume MB.

### 4.9 Intracellular ROS, GSH, mitochondrial potential, and lipid peroxidation

4T1 cells were incubated with ECT_Fe_ electrolytes (or pH-adjusted control media) and then assayed as follows. ROS: After 1 h treatment, cells were loaded with 10 μM DCFH-DA (Beyotime) for 30 min and analyzed for dichlorofluorescein fluorescence. Glutathione (GSH): After a 4-h treatment, intracellular GSH was measured using a GSH/GSSG assay kit (Beyotime). Mitochondrial membrane potential: Following a 4-h treatment, cells were stained with JC-1 (Beyotime) and imaged to capture both red and green fluorescent signals. Lipid peroxidation: Cells were treated for 4 h and then labeled with Lipoxite R590/G525 (AAT Bioquest); fluorescence imaging and flow cytometry were used to detect oxidized lipid signals.

### 4.10 Intratumoral ROS detection

Frozen tumor sections (collected ~4 h post-ECT) were stained with dihydroethidium (DHE) to detect superoxide/ROS *in situ* and imaged by fluorescence microscopy.

### 4.11 GPX4 expression analysis

In 4T1 cells, GPX4 protein expression was assessed by western blot (rabbit anti-GPX4 polyclonal, Beyotime; chemiluminescent detection on a Bio-Rad ChemiDoc). In tumor sections, GPX4 was examined by immunofluorescence staining and microscopy.

### 4.12 Cytokine quantification

Serum cytokines were measured by ELISA kits for IL-6 (ABclonal), TNF-α (ABclonal), IL-10 (ABclonal), and IFN-γ (Dakewe) according to manufacturers’ protocols.

### 4.13 *In vivo* bioluminescence imaging

4T1-luc tumor-bearing mice were imaged on days 1, 15, 17, 19, and weekly thereafter. D-luciferin (150 mg/kg) was injected intraperitoneally, and images were acquired 15 min later on an IVIS Spectrum (PerkinElmer). Luminescence was quantified as photons/second/cm²/sr using Living Image 4.4 software.

### 4.14 Toxicity and safety evaluations

Serum chemistry (ALB, ALP, ALT, AST, BUN, CREA, UA) was analyzed using an automated biochemical analyzer. Complete blood counts were performed on EDTA-anticoagulated blood using an automated hematology analyzer. Major organs (heart, liver, spleen, lung, kidney, muscle) from treated and model mice were fixed, H&E stained, and examined for pathology.

### 4.15 Statistical analysis

Data are presented as mean ± standard deviation (SD). Two-tailed unpaired *t*-tests were used for comparisons between two groups, and one-way ANOVA with appropriate *post hoc* tests was used for multi-group comparisons. Survival curves were analyzed by Kaplan–Meier method with log-rank test. *P* < 0.05 was considered statistically significant. Significance in figures is indicated as ns = not significant, *p < 0.05, **p < 0.01, ***p < 0.001, ****p < 0.0001.

## Data Availability

The original contributions presented in the study are included in the article/**Supplementary Material**. Further inquiries can be directed to the corresponding author.
