# Supplementary material for: Spatially programmed Fe and Pt electrode-driven electrochemistry reconditions the tumor microenvironment and sensitizes triple-negative breast cancer to PD-1 Blockade
Source: Front Oncol. 2026 Apr 29;16:1819923. doi: 10.3389/fonc.2026.1819923 (PMC13167493; doi:10.3389/fonc.2026.1819923)
Supplement: Supplementary file 1 [file DataSheet1.docx]

Supporting Information

**Spatially Programmed Fe and Pt Electrode-Driven Electrochemistry Reconditions the Tumor Microenvironment and Sensitizes Triple-Negative Breast Cancer to PD-1 Blockade**

Jia Qin, Yi He, Yueyao Yang, Yang Jiao, Zhipeng Liu, Yijie Xie, Wanling Lu, Ming Liu, Zhengyu Zhao, Dingjun Cai, Gang Wang*


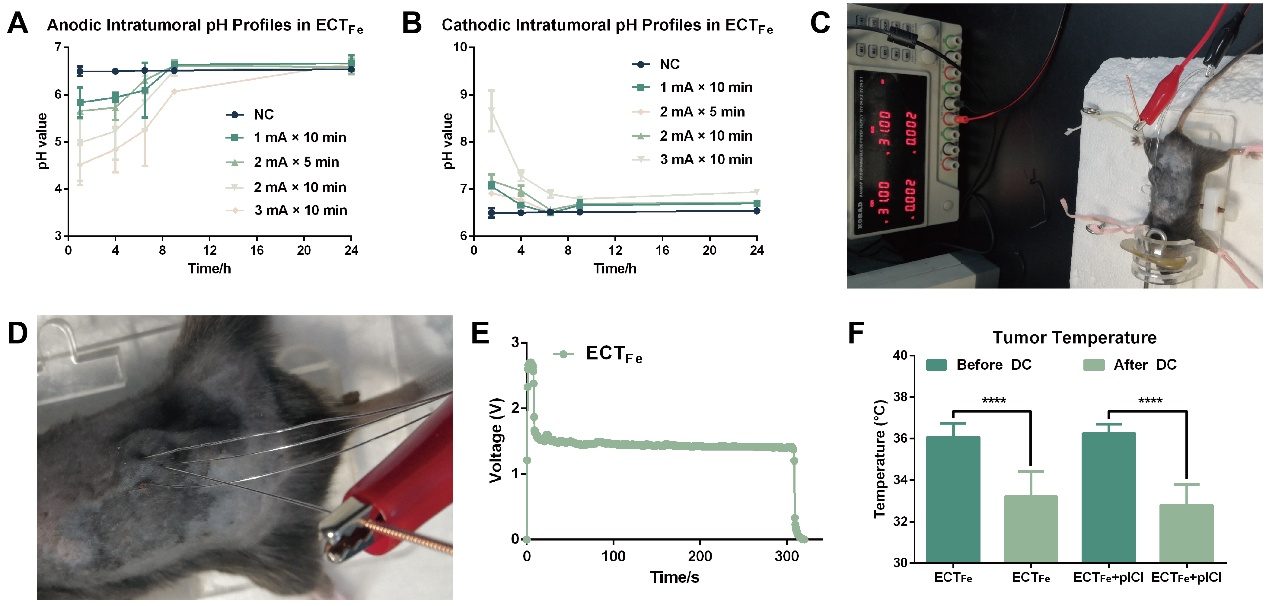


**Figure S1.** **pH dynamics and procedural data in the B16 melanoma after ECT_Fe_ treatment. (A, B) Time-course of pH changes measured at the iron (Fe) anode site (A) and platinum (Pt) cathode site (B) in B16 tumors after ECT_Fe_ treatment (n = 3 mice).** (**C**, **D**) Electrode placement of the Fe anode (within the tumor) and the Pt cathode (surrounding to the tumor). (**E**) Real-time voltage recording during ECT. (**F**) Tumor temperature pre/post ECT_Fe_, showing negligible heating (n ≥ 13 mice per group)**.** Data are presented as mean ± SD. Statistical significance was determined by one-way ANOVA**.** ns, not significant; **p* < 0.05; ***p* < 0.01; ****p* < 0.001; *****p* < 0.0001.


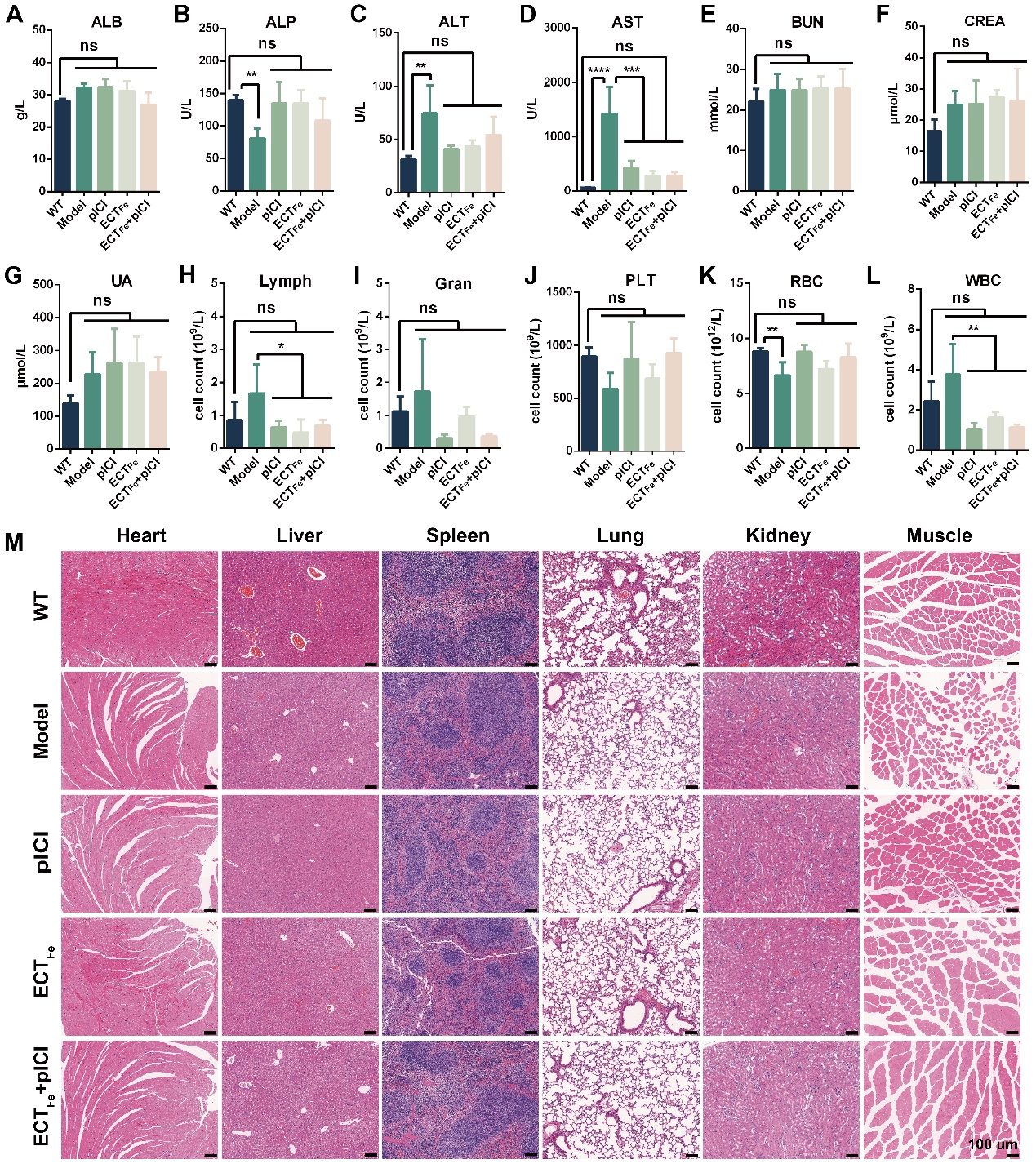


**Figure S2.** **Safety data of *in vivo* anti-B16 tumor treatment.** (A–L) Blood analysis: liver function biomarkers (**A**–**D**), renal function parameters (**E**–**G**), and complete blood count parameters (**H**–**L**). (**M**) Representative H&E-stained sections post-treatment, indicating no overt tissue damage. **Data are presented as mean ± SD.** n = 5 biologically independent mice per group. Statistical significance was determined by one-way ANOVA. ns, not significant; **p* < 0.05; ***p* < 0.01; ****p* < 0.001; *****p* < 0.0001. Scale bar, 100 μm.


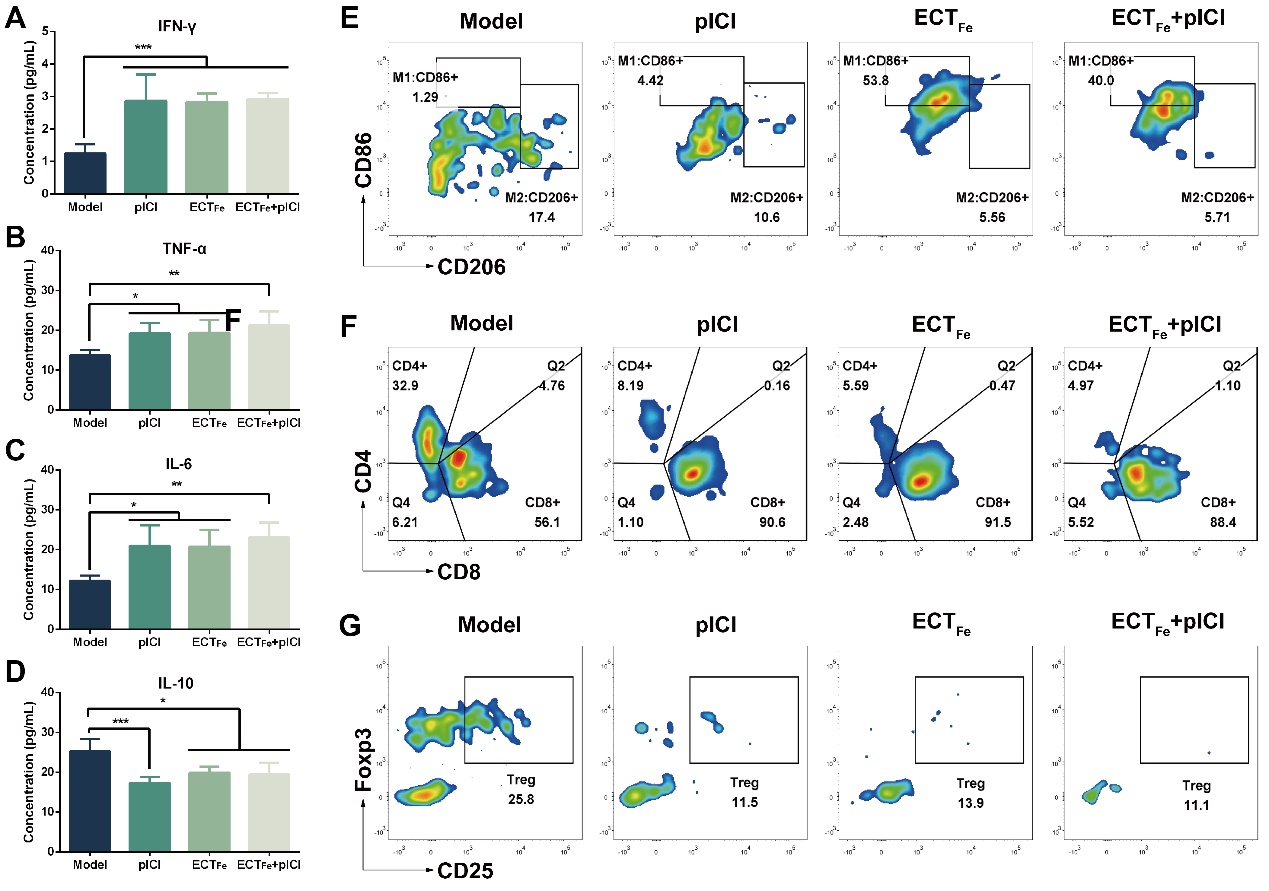


**Figure S3. Cytokine levels and flow cytometry gating in the B16 melanoma after ECT_Fe_ treatment.** (**A**–**D**) Serum levels of cytokines IFN-γ, TNF-α, IL-6, and IL-10 after treatment (n = 5 mice per group). **(E–G) Gating strategies for flow cytometric analysis used to identify: tumor-associated macrophage polarization (E); CD8⁺ T cells (F); and Tregs (G).** Data are presented as mean ± SD. One-way ANOVA was used for comparisons among groups. ns, not significant; **p* < 0.05; ***p* < 0.01; ****p* < 0.001; *****p* < 0.0001.


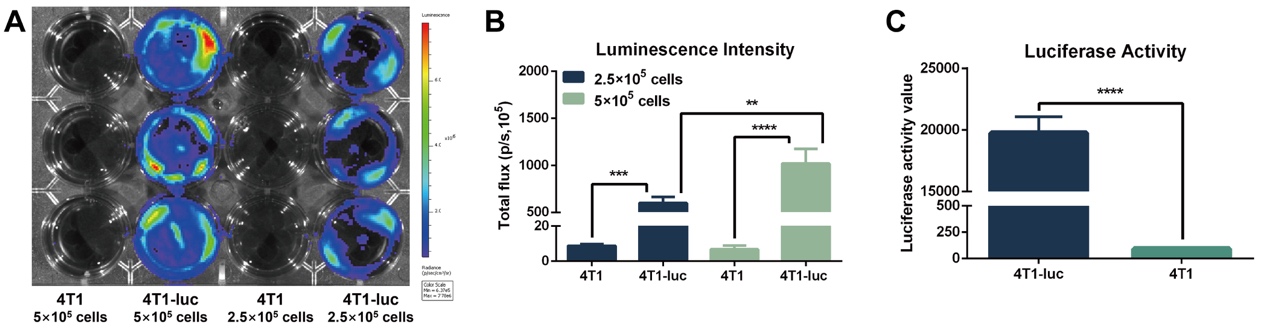


**Figure S4.** **Bioluminescence characterization of 4T1-luc cells *in vitro*.** (**A**) Bioluminescence imaging of cultured 4T1-luc cells after addition of luciferin. (**B**) Quantified luminescence intensity from 4T1-luc cell cultures, demonstrating the light output per cell number. (**C**) Luciferase enzyme activity in 4T1-luc cell lysates, confirming expression of the luciferase reporter. Data are presented as mean ± SD (n = 3 independent samples). One-way ANOVA was used for comparisons among groups. ns, not significant; **p* < 0.05; ***p* < 0.01; ****p* < 0.001; *****p* < 0.0001.


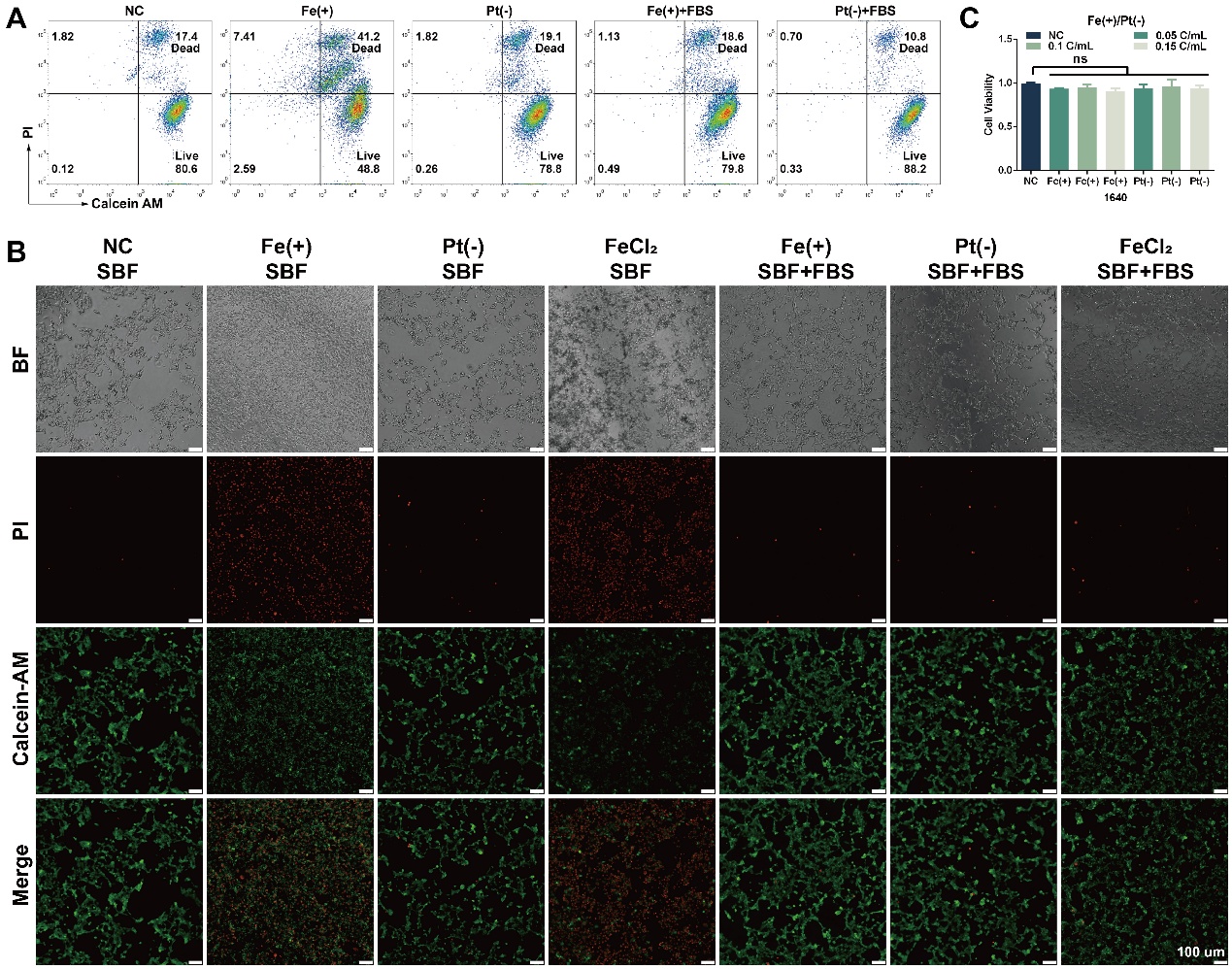


**Figure S5. Serum proteins (FBS) counteract the cytotoxicity of ECT_Fe_ *in vitro*.** (**A**) Flow cytometry quantification of cell death after a 4-h treatment with 0.15 C/mL ECT_Fe_ electrolyte, confirming extensive cell death under standard SBF conditions. (**B**) Live/dead fluorescence imaging of 4T1 cells (live: Calcein-AM, green; dead: PI, red) after a 4-h ECT_Fe_ electrolyte treatment (0.15 C/mL). (**C**) Cell viability after a 4-h treatment with 0.15 C/mL ECT_Fe_ electrolyte performed in RPMI-1640 medium (with serum), demonstrating that complete cell culture medium markedly reduces cytotoxicity compared to SBF. **Data are presented as mean ± SD.** n = 3 independent experiments. One-way ANOVA was used for comparisons. ns, not significant; **p* < 0.05; ***p* < 0.01; ****p* < 0.001; *****p* < 0.0001. Scale bar, 100 μm.


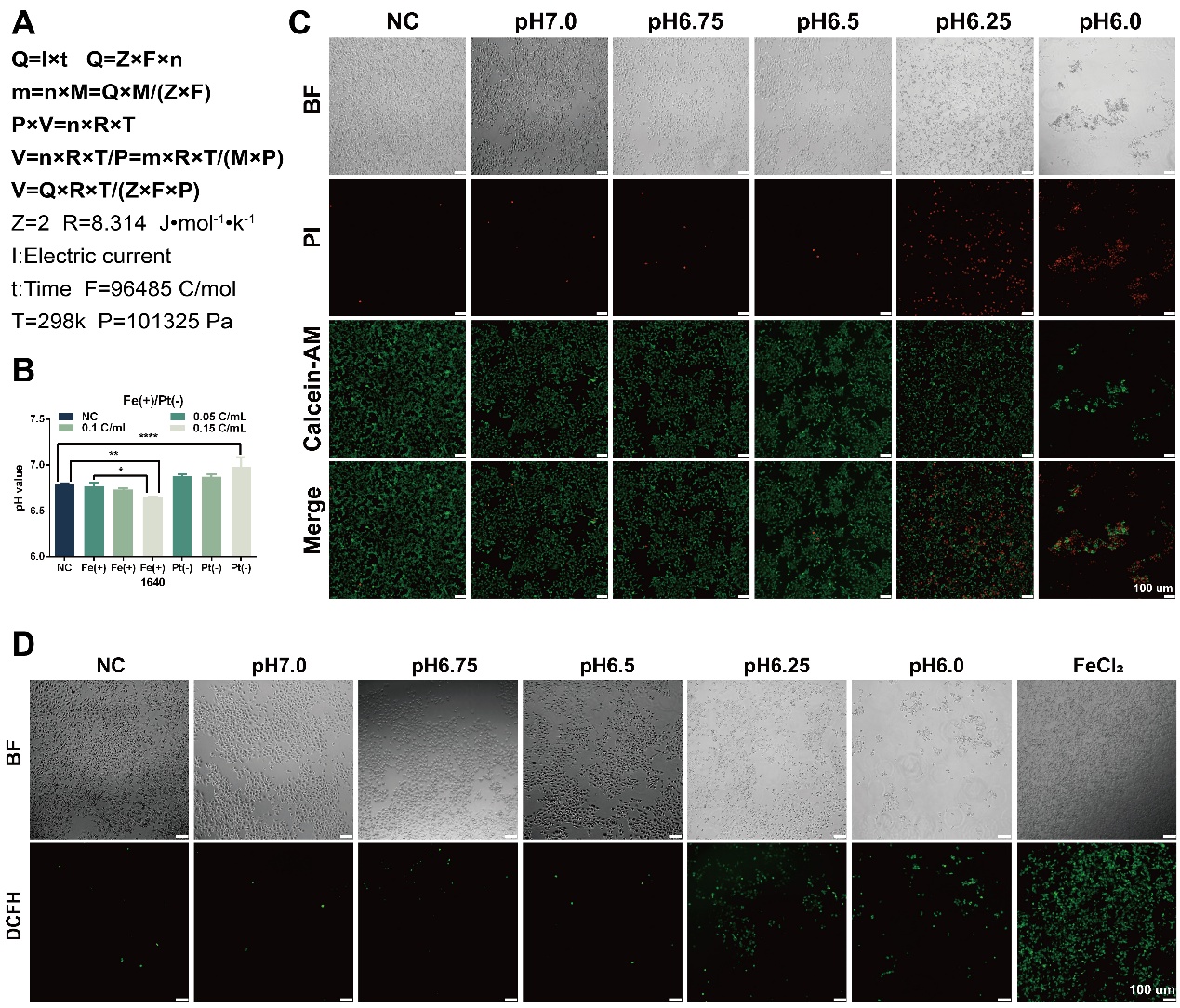


**Figure S6. pH changes in 1640 medium electrolyzed by ECT_Fe_ and the effects of low pH on 4T1 cells.** (**A**) Theoretical calculations of iron mass lost from the anode and H₂ gas volume produced at the cathode for a given charge passed (based on Faraday’s law and the ideal gas law). (**B**) Dose-dependent pH changes after ECT_Fe_ electrolysis in RPMI-1640. (**C**) Live/dead fluorescence imaging of 4T1 cells after 4 h exposure to acidic pH SBF, showing significant cell death caused by low pH alone. (**D**) Fluorescence imaging of intracellular ROS in 4T1 cells using the DCFH-DA probe after 4 h in the same acidic conditions as (C), indicating elevated ROS levels under low pH stress. **Data are presented as mean ± SD.** n = 3 independent experiments. One-way ANOVA was used for comparisons. ns, not significant; **p* < 0.05; ***p* < 0.01; ****p* < 0.001; *****p* < 0.0001. Scale bar, 100 μm.


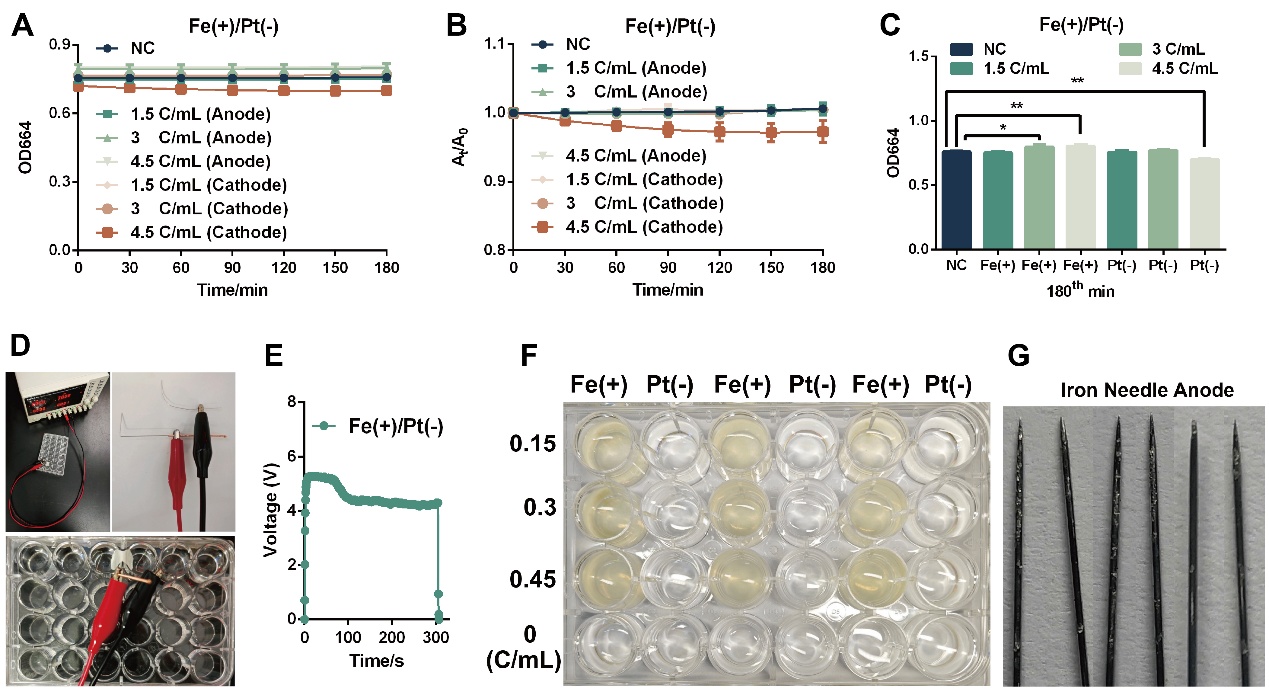


**Figure S7. Additional *in vitro* characterization of ECT_Fe_ on 4T1 cells (electrode corrosion and reactive species detection).** (A–C) Detection of anode-generated •OH and cathode-generated H₂ using an MB indicator in SBF: kinetics of time-dependent absorbance of MB at 664 nm (**A**); normalized curve by the ratio of absorbance (Aₜ/A₀) showing MB degradation (**B**); endpoint absorbance of MB at 180 min, representing final production of •OH and H₂ (**C**). (**D**) *In vitro* ECT_Fe_ electrolysis setup used for 4T1 cell treatment experiments. (**E**) Voltage profile (voltage *vs*. time) recorded during a constant-current ECT_Fe_ electrolysis in SBF. (**F**) Photograph of the SBF electrolyte after ECT_Fe_ electrolysis, showing discoloration and precipitation of iron corrosion products. (**G**) Photograph of the stainless-steel Fe anode after electrolysis, showing visible corrosion on its surface. **Data are presented as mean ± SD.** n = 3 independent experiments. One-way ANOVA was used for comparisons. ns, not significant; **p* < 0.05; ***p* < 0.01; ****p* < 0.001; *****p* < 0.0001.
